# Gabapentinoid Poisoning Presentations to Victorian Emergency Departments: A 15‐Year Trend Analysis

**DOI:** 10.5694/mja2.70275

**Published:** 2026-09-01

**Authors:** Bishaal Tej Gurung, Amy McNeilage, Angus Skeen, Tina Lam, Joanna F. Dipnall, Jane Hayman, Suzanne Nielsen, Ting Xia

**Affiliations:** ^1^ Monash Addiction Research Centre, Eastern Clinical School, Monash University Melbourne Victoria Australia; ^2^ School of Medicine, Monash University Melbourne Victoria Australia; ^3^ Faculty of Medicine and Health Sydney Medical School, University of Sydney Sydney New South Wales Australia; ^4^ School of Public Health and Preventive Medicine, Monash University Melbourne Victoria Australia; ^5^ School of Medicine, Institute for Mental and Physical Health and Clinical Translation, Deakin University Geelong Victoria Australia; ^6^ Victorian Injury Surveillance Unit, Accident Research Centre Monash University Melbourne Victoria Australia

**Keywords:** drug overdose, medical emergency services, pain

## Abstract

This study identified and characterised 1207 emergency department presentations related to gabapentinoid overdose in Victoria from 2009–2010 to 2023–2024. Presentation rates increased substantially since 2009–2010, and about half (49.5%; 597/1207) of all presentations were classified as intentional. Co‐ingestants were common, although 36.2% (437/1207) of presentations were reported to involve gabapentinoids alone.

## 1 Introduction

Gabapentinoids (pregabalin and gabapentin) are gamma‐aminobutyric acid (GABA) analogues predominantly used for analgesia. Australian prescribing increased more than eightfold within 6 years of their inclusion on the Pharmaceutical Benefits Scheme (PBS) in 2013 [1]. Concerns have emerged regarding potential gabapentinoid‐related harm, particularly when co‐ingested with other depressants such as opioids and benzodiazepines [2, 3]. Although fatal overdoses have been increasingly documented [4], non‐fatal poisonings remain poorly characterised because hospital coding systems do not reliably identify gabapentinoid‐related presentations. This study uses emergency department (ED) free‐text data to examine trends and circumstances of gabapentinoid poisoning presentations in Victoria.

## 2 Methods

This retrospective observational study uses data from July 2009 to June 2024 from the Victorian Emergency Minimum Dataset (VEMD), supplied by the Victorian Department of Health, which captures presentations to 41 public EDs across Victoria. In the absence of unique International Classification of Diseases and Related Health Problems, Tenth Revision, Australian Modification (ICD‐10‐AM) codes specific to gabapentinoid poisoning, relevant presentations were identified through a comprehensive text search of the ‘Description of event’ field, encompassing clinical features of toxicity or poisoning as well as relevant generic drug names, brand names and frequently occurring misspellings (Figure S1, Table S1). Two medical students (BG and AS) trained in clinical data coding independently reviewed all extracted records to identify gabapentinoid involvement in poisoning presentations, with an interrater agreement of 99.2% (Table S2). Identification was based on free‐text descriptions indicating overdose, toxicity or excessive ingestion, supplemented by hospital‐assigned cause poisoning classifications and relevant ICD‐10‐AM poisoning codes (T40, T509 and T659) (Tables S1–S3). Yearly rates per 100,000 population were calculated using Australian Bureau of Statistics population estimates. Chi‐square analyses compared presentation circumstances (intentional vs. unintentional poisoning, recorded as a structured field by hospital staff) by sex, age, triage level, alcohol involvement (identified through free‐text documentation and/or ICD‐10‐AM codes), and co‐ingested medications and/or substances. All analyses were conducted using Stata v17.0 (StataCorp). We reported our study in accordance with the Strengthening the Reporting of Observational Studies in Epidemiology (STROBE) guidelines (Table S4). This study was approved by the Monash University Human Research Ethics Committee (Approval No. 46332).

## 3 Results

**FIGURE 1:**
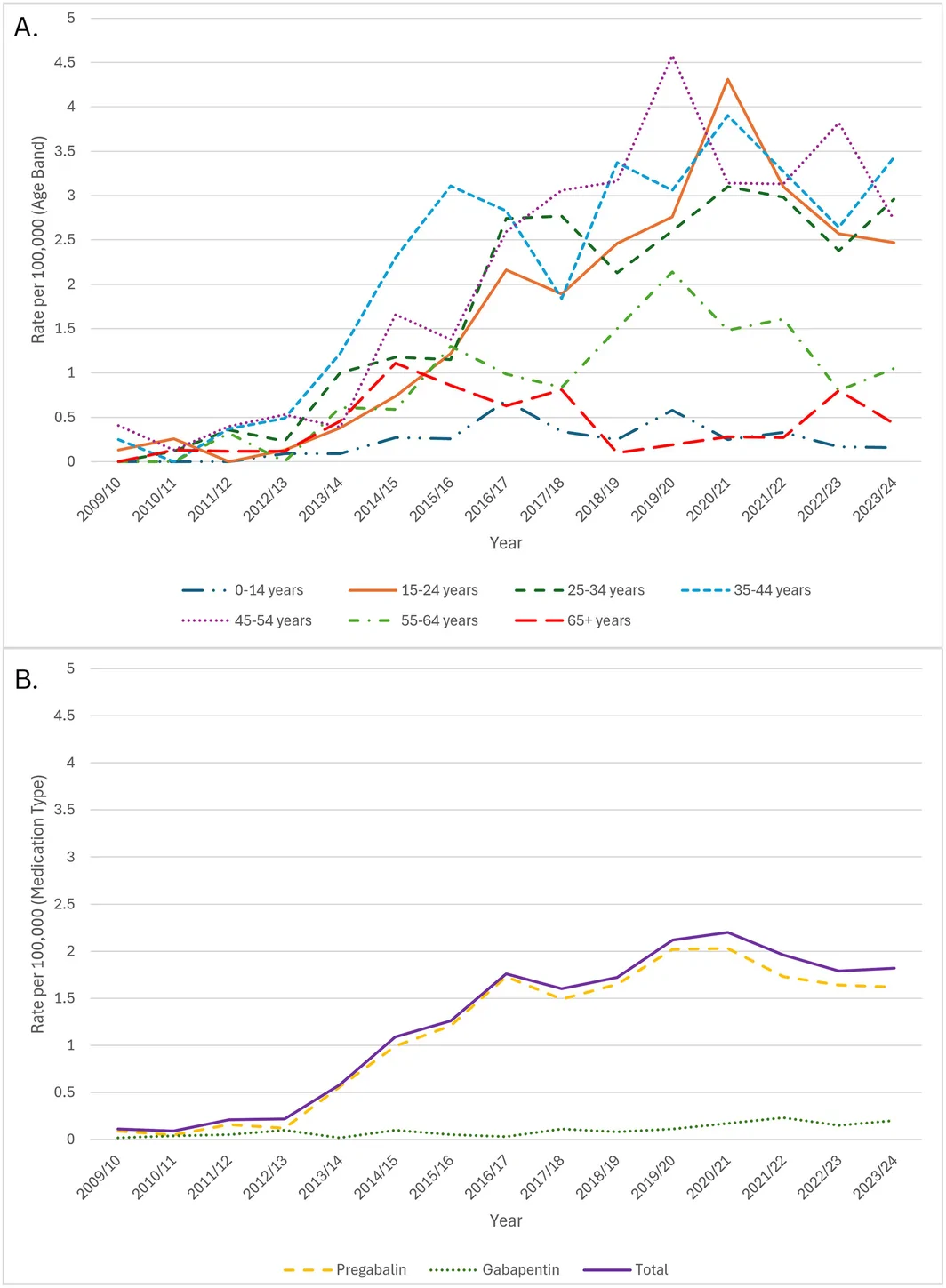
Trends in the rates of emergency department presentations involving gabapentinoid poisoning by age group (A) and medication type (B) in Victoria, Australia, from 2009–2010 to 2023–2024.

We identified 1207 ED presentations related to gabapentinoid poisoning, 56.0% (676/1207) of which involved females. The annual age‐specific rates increased steadily from 0.1 per 100,000 population in 2009–2010 to 2.2 per 100,000 population in 2020–2021, before stabilising thereafter. Presentations were most common among people aged 35–44 years (24.0%, 290/1207), followed by those aged 25–34 years (21.5%, 259/1207), with rates increasing across most age groups over time, particularly among adults aged 35–54 years (Figure 1A). Notably, a transient spike was observed among those aged 15–24 years in 2020–2021. Pregabalin consistently accounted for most presentations throughout the study period (Figure 1B). About half (49.5%, 597/1207) of all presentations were classified as intentional, and these were more common among females (55.3%, 374/676) than males (41.9%, 222/530; *χ* ^2^ = 21.5; *p* = 0.001) (Table 1). Intentional poisonings varied by age (*χ* ^2^ = 31.6; *p* = 0.001), with the highest proportions in those aged 55–64 years (60.4%, 58/96) and 45–54 years (55.7%, 142/255), and the lowest in those aged ≥ 65 years (22.6%, 14/62). Intentional poisoning was also more frequent among presentations triaged to resuscitation (41.7%, 35/84) and emergencies (54.9%, 242/441), whereas unintentional poisoning predominated in semi‐urgent and non‐urgent categories (72.8%, 67/92; *χ* ^2^ = 25.6; *p* = 0.001). Over a third (36.2%, 437/1207) of presentations involved gabapentinoids alone, while benzodiazepines and opioids were the most common co‐ingestants. Among presentations involving co‐ingested substances, more than half were classified as intentional.

**TABLE 1:** Emergency department presentations involving gabapentinoid poisoning by poisoning intent in Victoria, Australia, from 2009–2010 to 2023–2024.

	Intentional		Total (*N*, column %)
	Yes (*N*, row %)	No (unintentional and undetermined; *N*, row %)	
Total number of presentations	597	610	1207
Sex^a^, ^b^			
Female	374 (55.3%)	302 (44.7%)	676 (56.0%)
Male	222 (41.9%)	308 (58.1%)	530 (43.9%)
Age, years^b^			
0–14	17 (41.5%)	24 (58.5%)	41 (3.4%)
15–24	100 (49.0%)	104 (51.0%)	204 (16.9%)
25–34	114 (44.0%)	145 (56.0%)	259 (21.5%)
35–44	152 (52.4%)	138 (47.6%)	290 (24.0%)
45–54	142 (55.7%)	113 (44.3%)	255 (21.1%)
55–64	58 (60.4%)	38 (39.6%)	96 (8.0%)
≥ 65	14 (22.6%)	48 (77.4%)	62 (5.1%)
Triage level^b^			
Resuscitation	35 (41.7%)	49 (58.3%)	84 (7.0%)
Emergency	242 (54.9%)	199 (45.1%)	441 (36.5%)
Urgent	295 (50.0%)	295 (50.0%)	590 (48.9%)
Semi‐urgent or non‐urgent	25 (27.2%)	67 (72.8%)	92 (7.6%)
Co‐ingestant drug use^c^			
Yes	393 (51.0%)	377 (49.0%)	770 (63.8%)
No	204 (46.7%)	233 (53.3%)	437 (36.2%)
Alcohol involved			
Yes	79 (49.1%)	82 (50.9%)	161 (13.3%)
No	518 (49.5%)	528 (51.5%)	1046 (86.7%)

^a^ Sex was not specified for one presentation; as a result, totals within this category do not sum precisely.

^b^ *χ* ^2^ test, *p* < 0.01. For variables with more than two categories, this represents a statistically significant overall association across categories but does not indicate which individual categories differ from one another.

^c^ Substances covered under this category include opioids and benzodiazepines, along with any other substances documented in the ‘Description of event’ field. Benzodiazepines were involved in 25.0% (302/1207) of all cases, opioids were involved in 22.1% (267/1207), and both opioids and benzodiazepines were present in 9.0% (109/1207).

## 4 Discussion

This study demonstrates substantial growth in gabapentinoid‐related ED presentations in Victoria over the past 15 years, broadly paralleling increases in dispensing following subsidisation through the PBS [5]. Pregabalin accounted for most presentations, and almost half were coded as intentional. Consistent with existing literature, poisonings were concentrated in younger and middle‐aged populations despite higher dispensing rates in older adults [6]. Presentations involving co‐ingestants were more frequently triaged as higher acuity, consistent with evidence that gabapentinoid toxicity occurs primarily alongside other substances [2, 3]. One‐third of presentations involved no documented co‐ingestants. However, undocumented substance use cannot be excluded given the limitations of triage documentation, and these findings therefore do not confirm that single‐agent harm has occurred. This study may underestimate gabapentinoid‐related ED presentations, along with the presence of alcohol and other co‐ingestants, due to missing or non‐informative free‐text documentation. Identification of multi‐substance cases involved some subjective judgement, and intentionality or alcohol involvement may be under‐ascertained from structured fields. Free‐text records may not reflect final diagnoses, and observed trends may partly reflect changes in documentation practices over time. Despite these limitations, this study provides a comprehensive description of statewide ED presentations related to gabapentinoids over time, and our findings highlight the growing burden that gabapentinoids are placing on EDs, emphasising the need to manage co‐prescribing risks while strengthening support and risk assessment for those vulnerable to non‐medical use.

## Author Contributions

Bishaal Tej Gurung: Writing original draft and data analysis. Ting Xia, Suzanne Nielsen, Amy McNeilage and Tina Lam: Conceptualisation of the project. Angus Skeen: Conducting data validation and providing inputs to the original draft. Ting Xia, Suzanne Nielsen, Amy McNeilage, Tina Lam, Joanna F. Dipnall and Jane Hayman: Review, provide inputs and feedback on the writing. Ting Xia and Suzanne Nielsen contributed equally to this work as senior authors.

## Funding

This work was supported by the Australian National Health and Medical Research Council (NHMRC; GNT2037997). Suzanne Nielsen is the recipient of an NHMRC Investigator Grant (#2025894).

## Conflicts of Interest

The authors declare no conflicts of interest.

## Supporting information


**Data S1:** mja270275‐sup‐0001‐supinfo.pdf.

## Data Availability

The data used in this article are not available for distribution by the authors.
