# Supplementary material for: Gabapentinoid Poisoning Presentations to Victorian Emergency Departments: A 15‐Year Trend Analysis
Source: Med J Aust. 2026 Sep 1;224(9):e70275. doi: 10.5694/mja2.70275 (PMC13531248; doi:10.5694/mja2.70275)
Supplement: Supplementary file 1 [file MJA2-224-0-s001.pdf]

Supporting Information

Supplementary material

This appendix was part of the submitted manuscript and has been peer reviewed.
It is posted as supplied by the authors.

Appendix to: B. T. Gurung, A. McNeilage, A. Skeen, et al. Gabapentinoid Poisoning Presentations to Victorian Emergency Departments: A 15-Year Trend Analysis. *Medical Journal of Australia* 2026; doi: 10.5694/mja2.70275.

Figure S1. Flowchart of the extraction process

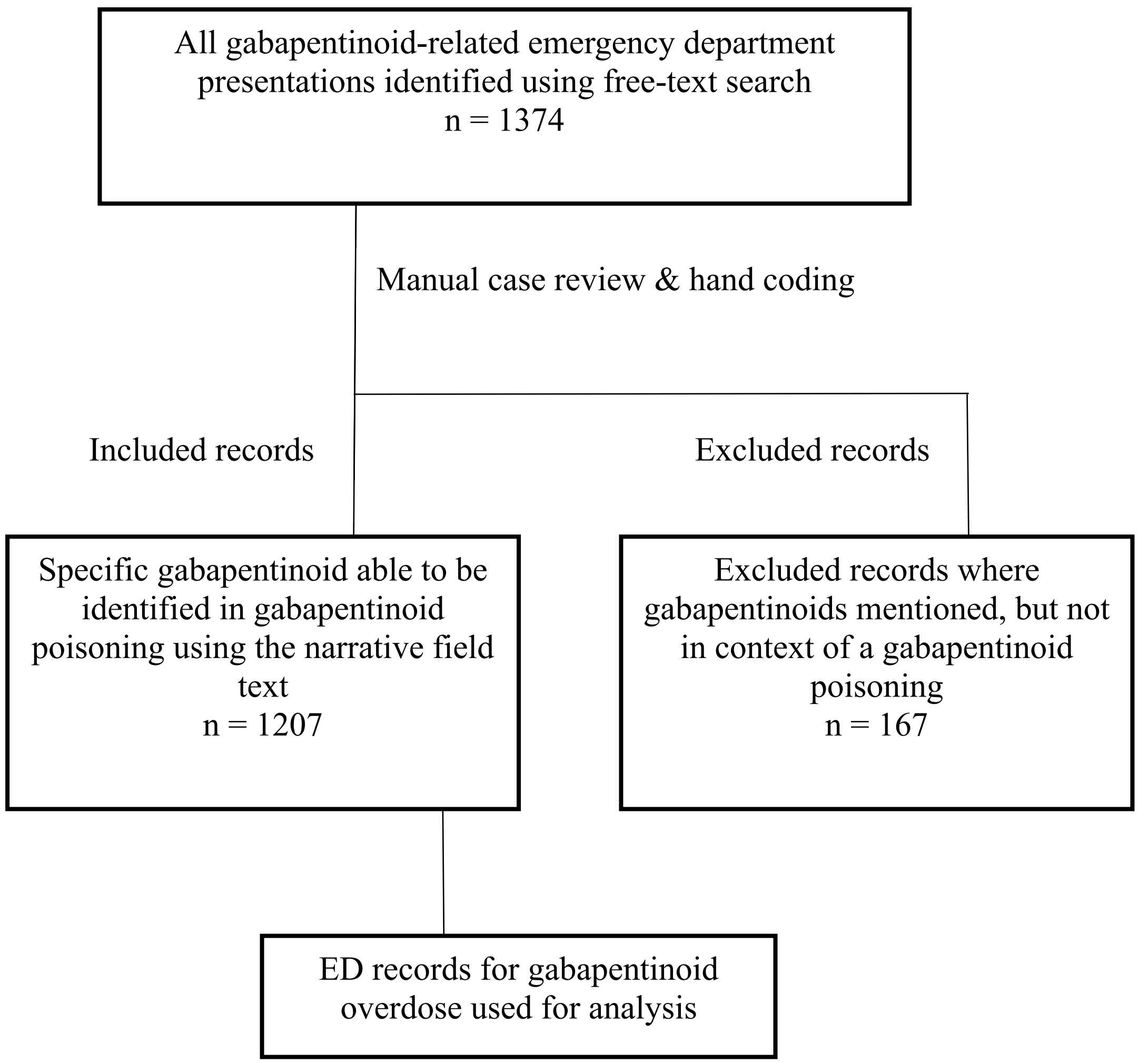

Table S1. Search terms and coding strategy[#]

<table>
<tr><th>Type of Search</th><th>Description</th></tr>
<tr><td>FREE-TEXT SEARCH FOR CASE IDENTIFICATION</td><td>Specific names of pharmaceutical gabapentinoids
[All gabapentinoid drug names including variations that include generic and brand names for the gabapentinoids of interest along with common misspellings as below]:
<table>
<tr><th>Correct Drug/Brand Spelling</th><th>Drug Misspellings</th><th>Brand Misspellings</th></tr>
<tr><td>Pregabalin/Lyrica</td><td>Pregamblin
Pregabolin
Pregalin
Pregabilin
Pregablin
Pregabalin</td><td>Lyrica
Lyrca
Lyri
Lyria
Lyric
Lyricax
Lyircs
Lyricca
Lyricsa
Lyrika
Lyriuka</td></tr>
<tr><td>Gabapentin/Neurontin</td><td>Gababapentin
Gabapentinnkda
Gabaentin
Gabap
Gabapanten
Gabapantin
Gabape
Gabapentinurofen
Gabapentinoid
Gabapentinpx
Gabapenton
Gabapertin
Gabatine
Ngabapentin
Gabapentine</td><td>Neurontin
Neuronton</td></tr>
</table>
AND
Specific mention of Poisoning/overdose, such as ‘overdose,’ ‘od,’ ‘o/d,’ ‘poisoning,’ ‘drug abuse,’ ‘toxicity,’ ‘poison,’ ‘self harm,’ or ‘suicide’. OR

Broad ICD-10-AM codes suggested drug poisoning including T40 (‘poisoning by narcotics and psychodysleptics’), T509 (‘poisoning by, adverse effect of, and underdosing of other and unspecified drugs, medicaments, and biological substances’), or T659 (‘toxic effect of unspecified substance’); or VEMD internal coding of broad cause group was coded under</td></tr>
</table>

	‘Poisoning-medication (code 17)’ and ‘Poisoning- other or unspecified (code 18)’.
POLYPHARMACY / CO-INGESTION CODING (for poisoning cases only)	For each poisoning case, code presence of other substances into predefined categories:<br>• **O (opioids):** any opioid mentioned (e.g. heroin, methadone, oxycodone, codeine, etc.)<br>• **B (benzodiazepines):** any benzodiazepine mentioned (e.g. diazepam, temazepam, etc.)<br>• **BOTH:** both opioids AND benzodiazepines mentioned<br>• **E (other):** other co-ingestants only (alcohol, stimulants, antipsychotics, etc.)<br>• **N (none):** no other substance mentioned

[#] The initial search strategy included the identification of specific drug names, brand names, and common misspellings. Cases were subsequently flagged as poisoning or overdose if either: (1) the ‘Description of Event’ field contained terms such as ‘overdose,’ ‘od,’ ‘o/d,’ ‘poisoning,’ ‘drug abuse,’ ‘toxicity,’ ‘poison,’ ‘self harm,’ or ‘suicide’; or (2) broad ICD-10-AM codes suggested drug poisoning including T40 (‘poisoning by narcotics and psychodysleptics’), T509 (‘poisoning by, adverse effect of, and underdosing of other and unspecified drugs, medicaments, and biological substances’), or T659 (‘toxic effect of unspecified substance’); (3) VEMD internal coding of broad cause group was coded under ‘Poisoning-medication (code 17)’ and ‘Poisoning- other or unspecified (code 18)’.

Table S2. Interrater Reliability

Identification of gabapentinoid overdose*	Type of gabapentinoid	Co-ingestants*
99.2%	100%	93.7%

* In cases where there was discrepancy between classifications during manual validation, a consensus was reached between both reviewers to determine the final decision.

Table S3. Manual coding framework applied to mock case examples

Example No.	Free text	Identification of gabapentinoid overdose	Type of gabapentinoid	Co-ingestants
1	Pregabalin OD	Yes	Pregabalin	N (None)
2	Brought in following exacerbation of back pain, usually takes paracetamol, gabapentin and ibuprofen	No	N/A	N/A
3	Overdose on neuronton, diaz, panadeine forte	Yes	Gabapentin	BOTH (Opioids and Benzodiazepine)
4	Patient took 50x75mg pregabalin tabs, biba gcs 13	Yes	Pregabalin	N (None)
5	Patient presented with atraumatic leg pain, given pregabalin with minimal effect	No	N/A	N/A
6	Biba drowsy after they accidentally took too many of their prescribed lyrica tabs	Yes	Pregabalin	N (None)
7	Gabapentinoid OD	Yes	Unspecified	N (None)

Table S4. STROBE Statement—checklist of items that should be included in reports of observational studies

	Item No.	Recommendation	Included in Study?
**Title and abstract**	1	(*a*) Indicate the study's design with a commonly used term in the title or the abstract	**Yes (see Abstract)**
		(*b*) Provide in the abstract an informative and balanced summary of what was done and what was found	Yes (see Abstract)
**Introduction**			
Background/rationale	2	Explain the scientific background and rationale for the investigation being reported	Yes (see Introduction)
Objectives	3	State specific objectives, including any prespecified hypotheses	Yes (see Introduction)
**Methods**			
Study design	4	Present key elements of study design early in the paper	Yes (see Methods)
Setting	5	Describe the setting, locations, and relevant dates, including periods of recruitment, exposure, follow-up, and data collection	Yes (see Methods)
Participants	6	(*a*) *Cohort study*—Give the eligibility criteria, and the sources and methods of selection of participants. Describe methods of follow-up<br>*Case-control study*—Give the eligibility criteria, and the sources and methods of case ascertainment and control selection. Give the rationale for the choice of cases and controls<br>*Cross-sectional study*—Give the eligibility criteria, and the sources and methods of selection of participants	Yes (see Methods)
		(*b*) *Cohort study*—For matched studies, give matching criteria and number of exposed and unexposed<br>*Case-control study*—For matched studies, give matching criteria and the number of controls per case	N/A
Variables	7	Clearly define all outcomes, exposures, predictors, potential confounders, and effect modifiers. Give diagnostic criteria, if applicable	Yes (See Methods)
Data sources/ measurement	8*	For each variable of interest, give sources of data and details of methods of assessment (measurement). Describe comparability of assessment methods if there is more than one group	Yes (Methods)
Bias	9	Describe any efforts to address potential sources of bias	Yes (Discussion)
Study size	10	Explain how the study size was arrived at	Yes (Methods and Supplementary)
Quantitative variables	11	Explain how quantitative variables were handled in the analyses. If applicable, describe which groupings were chosen and why	Yes (Methods)
Statistical methods	12	(*a*) Describe all statistical methods, including those used to control for confounding	Yes (See Methods)
		(*b*) Describe any methods used to examine subgroups and interactions	Yes (Methods)
		(*c*) Explain how missing data were addressed	N/A

		(*d*) *Cohort study*—If applicable, explain how loss to follow-up was addressed<br>*Case-control study*—If applicable, explain how matching of cases and controls was addressed<br>*Cross-sectional study*—If applicable, describe analytical methods taking account of sampling strategy	N/A
		(*e*) Describe any sensitivity analyses	N/A
**Results**			
Participants	13*	(a) Report numbers of individuals at each stage of study—eg numbers potentially eligible, examined for eligibility, confirmed eligible, included in the study, completing follow-up, and analysed	Yes (See Supplementary Figure S1)
		(b) Give reasons for non-participation at each stage	Yes (See Supplementary Figure S1)
		(c) Consider use of a flow diagram	Yes (See Supplementary Figure S1)
Descriptive data	14*	(a) Give characteristics of study participants (eg demographic, clinical, social) and information on exposures and potential confounders	Yes (See Results)
		(b) Indicate number of participants with missing data for each variable of interest	Yes (See Results)
		(c) *Cohort study*—Summarise follow-up time (eg, average and total amount)	N/A
Outcome data	15*	*Cohort study*—Report numbers of outcome events or summary measures over time	Yes (See Results)
		Case-control study—Report numbers in each exposure category, or summary measures of exposure	N/A
		Cross-sectional study—Report numbers of outcome events or summary measures	N/A
Main results	16	(*a*) Give unadjusted estimates and, if applicable, confounder-adjusted estimates and their precision (eg, 95% confidence interval). Make clear which confounders were adjusted for and why they were included	Yes (See Results)
		(*b*) Report category boundaries when continuous variables were categorized	Yes (See Results)
		(*c*) If relevant, consider translating estimates of relative risk into absolute risk for a meaningful time period	N/A
Other analyses	17	Report other analyses done—eg analyses of subgroups and interactions, and sensitivity analyses	Yes (See Results)
**Discussion**			
Key results	18	Summarise key results with reference to study objectives	Yes (See Discussion)
Limitations	19	Discuss limitations of the study, taking into account sources of potential bias or imprecision. Discuss both direction and magnitude of any potential bias	Yes (See Discussion)
Interpretation	20	Give a cautious overall interpretation of results considering objectives, limitations, multiplicity of analyses, results from similar studies, and other relevant evidence	Yes (See Discussion)
Generalisability	21	Discuss the generalisability (external validity) of the study results	Yes (See Discussion)
**Other information**			

Funding	22	Give the source of funding and the role of the funders for the present study and, if applicable, for the original study on which the present article is based	Yes (see Funding)
